# Acute Promyelocytic Leukemia Presenting with Severe Marrow Fibrosis

**DOI:** 10.1155/2015/826894

**Published:** 2015-07-12

**Authors:** Harsh Shah, Carol Bradford, Hamid Sayar

**Affiliations:** ^1^Indiana University Simon Cancer Center, Indianapolis, IN, USA; ^2^Eli Lilly and Company, Indianapolis, IN, USA

## Abstract

We report a case of acute promyelocytic leukemia (APL) presenting with severely fibrotic marrow. There are four other reports of similar cases in the literature. Our patient was treated with All-Transretinoic Acid- (ATRA-) containing induction chemotherapy, followed by consolidation and maintenance therapy. He achieved a complete morphologic remission with adequate count recovery in a timely fashion, and later a molecular remission was documented. The patient remains in molecular remission and demonstrates normal blood counts now more than 4 years after induction. Since the morphological appearance may not be typical and the bone marrow may not yield an aspirate for cytogenetic analysis, awareness of such entity is important to make a correct diagnosis of this potentially curable disease.

## 1. Introduction

Variable levels of focal or diffuse reticulin fibrosis are detected in the bone marrow samples of up to 30% of patients with acute leukemia [1]. However, there are only four case reports in the literature of acute promyelocytic leukemia (APL) presenting with severe reticulin bone marrow fibrosis [2–5]. It is important to differentiate APL with fibrosis from other types of fibrotic acute myeloid leukemia (AML) in order to provide the most appropriate therapy and proper surveillance. Here, we report a case of 48-year-old previously healthy male who presented with APL and severe bone marrow reticulin fibrosis.

## 2. Case Report

In June 2010, a 48-year-old male was admitted to the hospital with extreme fatigue and severe pancytopenia with hemoglobin of 6.0 g/dl, platelet count of 19 × 10^9^/L, and white blood cell count of 0.6 × 10^9^/L (88% lymphocytes, 8% neutrophils, and 4% monocytes). The patient did not have remarkable findings on physical examination such as hepatomegaly or splenomegaly. An initial bone marrow examination resulted in a “dry tap” and a core biopsy showed hyperplasia with severely increased reticulin fibrosis and less than 5% blasts on a CD34 stain. A number of atypical myeloperoxidase-positive cells were also reported. Cytogenetics test was unsuccessful due to nonviability of cells. The patient was referred to our institution for further workup and treatment.

**Figure 1:**
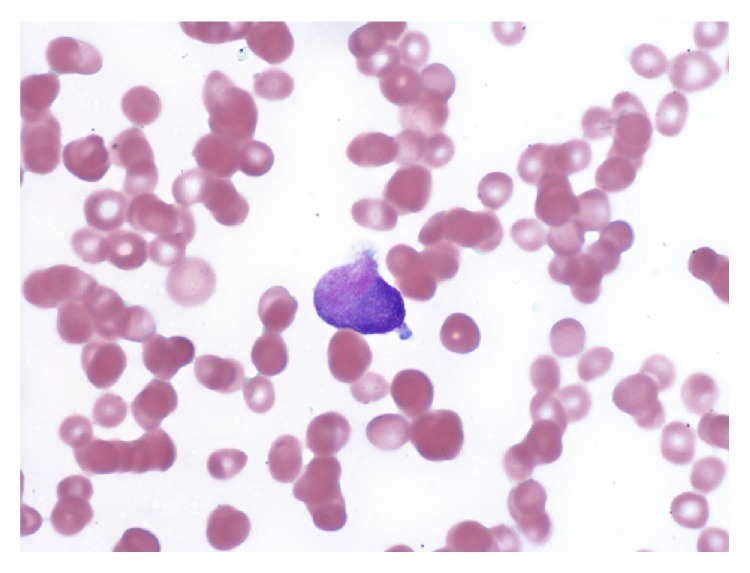
Promyelocyte in the peripheral blood smear.

**Figure 2:**
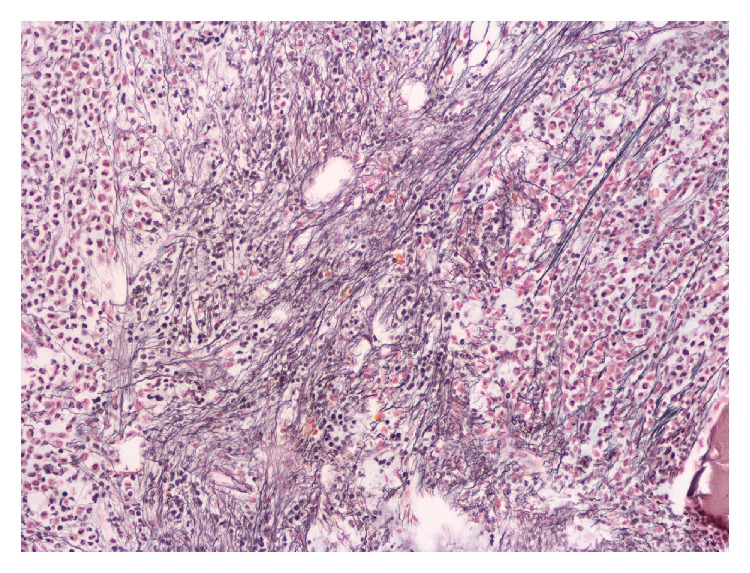
Bone marrow reticulin stain shows severe fibrosis.

**Figure 3:**
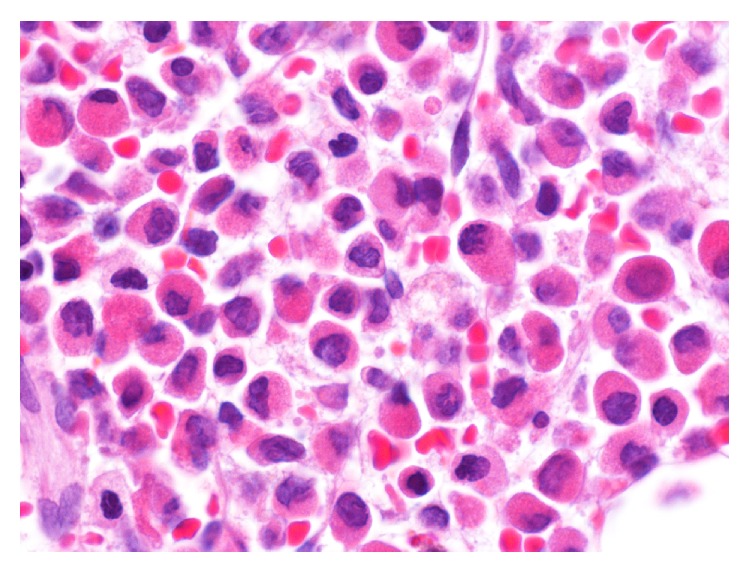
Bone marrow biopsy showing increased granular cells.

Initially with a diagnosis of myeloid neoplasm with myelofibrosis, the patient was considered a candidate for allogeneic stem cell transplantation. However, a careful review of the peripheral blood smear on a follow-up visit revealed a blast containing multiple Auer rods and rare promyelocytes, raising suspicion for APL (Figure 1). There were also occasional tear drop red blood cells present, but no nucleated red cells. A fluorescence in situ hybridization (FISH) analysis on the peripheral blood demonstrated PML/RARA rearrangement. A repeat bone marrow examination was also attempted. The bone marrow aspirate yielded a dry tap again, but the core biopsy indicated severely increased reticulin fibrosis and focal increase of CD34 positive cells (5–10%) (Figure 2). A population of atypical myeloperoxidase- and CD117-positive granular cells comprising 75–80% of nucleated marrow cells were also observed (Figure 3). Chromosome analysis on the core biopsy sample confirmed t(15;17)(q24;q21.1).

In July 2010, the patient received induction chemotherapy with Idarubicin and ATRA per AIDA-2000 protocol [6] and demonstrated adequate blood count recovery starting around day 28 of induction. Postinduction bone marrow aspiration was a dry tap, but the core biopsy showed no evidence of leukemic blasts or promyelocytes. There was persistent severe reticulin fibrosis in the marrow. A FISH study for PML/RARA on this biopsy was negative, but PCR test of peripheral blood detected a low level of positivity for PML/RARA transcripts. He was subsequently treated with 2 cycles of consolidation therapy with Idarubicin and ATRA and experienced normal count recovery each time. A postconsolidation PCR analysis on the peripheral blood did not show detectable PML/RARA transcripts. The patient received 2 years of maintenance oral chemotherapy and ATRA from December 2010 to December 2012. He has maintained normal blood counts with no tear drop or nucleated red cells on morphology and has remained in molecular remission to date, more than 4 years after achievement of remission.

## 3. Discussion

Despite frequent observation of some degrees of fibrosis in the bone marrow of patients with AML, there are only 4 case reports of severe marrow fibrosis in the setting of APL. Marrow fibrosis is thought to be secondary to activation of normal fibroblasts by cytokines secreted from CD34+/HLA-DR+ leukemic blasts, markers that are usually not expressed by APL cells [3]. Per the immunostaining, our patient had only limited number of CD34-expressing blasts in the marrow. It is also questionable why many patients with AML do not develop marrow fibrosis despite abundance of CD34+/HLA-DR+ leukemic blasts.

It is usually a concern that leukemic patients with marrow fibrosis may experience delayed or no marrow recovery following myelosuppressive induction therapy. However, Islam et al. reported, based on review of 34 cases, that an increase in marrow fiber content at diagnosis did not affect hematopoietic regeneration following treatment of acute leukemia [7]. In case of APL, the existing case reports indicate adequate bone marrow regeneration after induction chemotherapy. Our patient also demonstrated blood count recovery to normal levels in a timely manner following his induction therapy. Therefore, presence of marrow fibrosis of any severity should not be a prohibiting factor to administer standard doses of induction chemotherapy with curative intent to patients with APL.

Manoharan et al. reported that survival in acute leukemia patients with marrow fibrosis correlated with remission status rather than the marrow reticulin content [8]. Reported patients with APL and bone marrow fibrosis were treated with different regimens, but all received ATRA as part of their therapeutic regimen. All reported patients achieved a complete morphologic remission following induction therapy. Two cases demonstrated molecular remission with undetectable PML/RARA transcripts. It is unclear as to whether the two other patients ever experienced a molecular remission or not. Out of 4 reported cases, the disease relapsed in one after 19 months of diagnosis, but the three others remained in remission to the time of report. One patient was in remission after 21 months, another was still on maintenance therapy, and the duration of follow-up was not reported for the third patient in remission. Our patient achieved both morphologic remission and cytogenetic remission documented by a bone marrow examination on day 36 of induction chemotherapy. He also achieved a complete molecular remission documented by peripheral blood PCR analysis following completion of his courses of consolidation therapy. The patient remains in molecular remission now more than 4 years after induction chemotherapy. To our knowledge, this is the longest remission duration reported in the literature in patients with APL and marrow fibrosis.

Finally, there are discrepant reports regarding disappearance of marrow fibrosis following achievement of remission in patients with fibrotic AML. Among reported APL patients, the fibrosis was still present at time of postinduction bone marrow examination in all patients. It disappeared later in 2 cases and was not evaluated in the other two. Our patient also demonstrated persistent fibrosis in his postinduction bone marrow on day 36. A repeat bone marrow examination was not attempted later; therefore the status of his marrow fibrosis remains unknown. He has, however, maintained normal blood counts since achievement of morphologic remission.

## 4. Conclusion

Bone marrow fibrosis has only rarely been reported in patients with APL. It is unclear why the incidence of fibrosis is much smaller in APL compared to other subtypes of AML, but overproduction of fibrosis may be related to cytokine effect of CD34- and HLA-DR-expressing blasts which are more abundant in other subtypes than APL. Based on limited number of reports it appears that these patients respond to ATRA-containing therapeutic regimens similar to nonfibrotic APL. Moreover, they experience adequate and timely marrow recovery as well as lengthy molecular remission. It is important for practicing hematologists and hematopathologists to be aware of such entity since a correct diagnosis of APL with early incorporation of ATRA in the therapeutic regimen can offer a high chance of cure.
